# Differential expression of ferroptosis-related proteins in urinary exosomes: potential indicators for monitoring acute gout attack

**DOI:** 10.3389/fmolb.2024.1476631

**Published:** 2024-11-01

**Authors:** Jitu Wang, Yubin Lin, Na Liu, Mei Hu, Man Zhang

**Affiliations:** ^1^ Clinical Laboratory Medicine, Beijing Shijitan Hospital, Capital Medical University, Beijing, China; ^2^ Beijing Key Laboratory of Urinary Cellular Molecular Diagnostics, Beijing, China; ^3^ Institute of Regenerative Medicine and Laboratory Technology Innovation, Qingdao University, Qingdao, China

**Keywords:** acute gout attack, gout, urine exosomes, ferroptosis, biomarker

## Abstract

**Background:**

Gout is the most prevalent form of inflammatory arthritis, characterized by significant pain during acute episodes. Current diagnostic and monitoring techniques are invasive and fail to predict the onset of acute attacks. Recent studies have implicated ferroptosis-related proteins in the pathogenesis of inflammation and gout; however, their clinical relevance in gout patients remains largely unexplored. This study aimed to evaluate the expression of these proteins in urinary exosomes from gout patients and to investigate their potential as noninvasive biomarkers.

**Methods:**

Utilizing data-independent acquisition (DIA) mass spectrometry and advanced bioinformatics techniques, we assessed the expression of ferroptosis-related proteins in the urinary exosomes of three groups: acute gout patients (AD group), intermittent gout patients (ID group), and normal controls (NC group). We constructed receiver operating characteristic (ROC) curves to determine the clinical utility of these proteins in monitoring acute gout attacks.

**Results:**

Our analysis of urinary exosome proteomics identified 13 ferroptosis-related proteins. Notably, in comparison to the ID group, the proteins ACSL4, VDAC2, GPX4, and GSS were significantly upregulated in the AD group. ROC curve analysis revealed that the presence of ACSL4, VDAC2, and GPX4 in urinary exosomes possesses substantial predictive value for acute gout attacks.

**Conclusion:**

In patients with gout, numerous protein alterations occur within urinary exosomes. Specifically, changes in ferroptosis-related proteins such as ACSL4, VDAC2, GPX4, and GSS may serve as promising biomarkers for the monitoring of acute gout attacks.

## 1 Introduction

Gout is an inflammatory disease caused by high levels of uric acid in the blood, leading to various forms of joint and tissue damage. Urate crystals can deposit in different body tissues, such as the synovium, bursae, and cartilage, causing acute inflammation. Gout is the most common type of inflammatory arthritis (Neogi et al., 2015). With rapid economic development and significant lifestyle changes, the prevalence of hyperuricemia and gout has increased significantly worldwide (Butler et al., 2021). In China, hyperuricemia affects 13.3% of the population, and the prevalence of gout is 1%–3%. The incidence in males is 2–5 times higher than in females, and the overall prevalence is rising year by year (Kuo et al., 2015; Liu et al., 2015). With the increasing incidence of gout, research into its biomarkers has become a major focus. More researchers are exploring the mechanisms of gout and using bioinformatics analysis to identify effective biomarkers for the disease (Song et al., 2024; Goldberg et al., 2017; Rasheed et al., 2016).

Patients may have no symptoms before the acute phase of gout. During an acute gout attack, patients are often awakened at night by severe joint pain. Gout has a high recurrence rate, and currently, there are no methods to predict its onset. Recurrent gout attacks can lead to gouty arthritis and structural joint damage. The inflammation associated with gout can also contribute to atherosclerosis and promote thrombosis (Jin et al., 2012). Additionally, acute gout attacks can cause complications such as diabetes, hyperlipidemia, metabolic syndrome, and increase the risk of cardiovascular and cerebrovascular diseases (Chen et al., 2007; Wang et al., 2020; Thanassoulis et al., 2010; Zhu et al., 2012).

Currently, the diagnosis and monitoring of gout primarily involve tests such as serum uric acid levels, joint fluid analysis, and gouty tophus composition analysis. These tests are invasive and cannot predict the acute phase of a gout attack. Repeated invasive examinations can cause additional distress to patients who are already experiencing pain. Urine, as an ultrafiltrate of blood, retains significant biological information found in plasma. The inhibitory effects of high-abundance proteins are not pronounced, making it easier to identify low-abundance proteins that could serve as potential disease markers in urine (Chen et al., 2013; Fu et al., 2016). Urine collection is a non-invasive procedure that is simple to perform, repeatable, and does not increase patient discomfort. Exosomes are extracellular vesicles derived from endosomal compartments involved in intercellular communication and signal transduction. Urinary exosomes can reflect the physiological and pathological states of their source cells and have the potential to serve as biomarkers, making them excellent carriers for studying gout biomarkers.

Iron is an essential trace element in the human body, widely involved in various biological processes, including DNA synthesis, erythropoiesis, and mitochondrial biosynthesis (Gozzelino and Arosio, 2016). Many studies have shown that ferroptosis is immunogenic and closely related to the onset and progression of inflammation (Kim et al., 2019; Proneth and Gonrad, 2019). Intracellular iron overload is crucial for ferroptosis (Zhou et al., 2018). Iron overload can increase serum uric acid levels, thereby heightening the risk of elevated blood uric acid and gout attacks (Richette and Latourte, 2018; Fatima et al., 2018). The underlying mechanism and the relationship between ferroptosis and gout has gradually become a research hotspot (Guo et al., 2024).

In this study, we analyzed changes in ferroptosis-related proteins in urinary exosomes from patients experiencing acute gout attacks and those in the intercritical period. We evaluated the ability of differentially expressed ferroptosis-related proteins in urinary exosomes to monitor acute gout attacks, providing a theoretical basis for research on non-invasive biomarkers for the diagnosis and monitoring of acute gout.

## 2 Materials and methods

### 2.1 Study subjects

Between August 2021 and February 2022, a total of 24 male gout patients with elevated serum uric acid levels were recruited from Beijing Shijitan Hospital, Capital Medical University. This cohort comprised 12 patients experiencing acute gout attacks (AD group) and 12 patients with non-acute gout attacks (ID group). Additionally, 12 age-matched healthy individuals were included as the normal control (NC) group. All gout patients fulfilled the 2015 American College of Rheumatology/European League Against Rheumatism (ACR/EULAR) classification criteria for gout. An acute gout attack was characterized by a score of ≥8 and the presence of swelling, pain, and/or tenderness in peripheral joints or bursa, while patients with non-acute gout attacks had a score of <8 and a documented history of gout. The diagnosis of hyperuricemia adhered to the 2019 Chinese guidelines for the diagnosis and treatment of the condition. All participants were free from hematuria, proteinuria, hyperlipidemia, ketosis, and other forms of arthritis, urological diseases, or tumors. To control for potential confounding factors, participants’ smoking history, alcohol consumption, and adherence to a high-purine diet were meticulously assessed.

### 2.2 Sample collection

Morning urine samples (30 mL) were collected from each subject and subsequently centrifuged at 4°C. The samples were processed initially at 1,500 g for 10 min, followed by a second centrifugation at 10,000 g for 30 min to effectively remove cellular components and debris. The supernatant was then filtered using a 0.22 μm pore size, 33 mm diameter filter (Millipore, SLGVR33RB). To concentrate the urine, an ultrafiltration tube (Millipore, UFC910024) was employed, and exosomes were isolated from the concentrated samples using a size exclusion column (qEV10/35nm, IZON, Shanghai, China) (Foers et al., 2018; Guo et al., 2021). The collected exosomes were finally resuspended in approximately 1 mL of phosphate-buffered saline (PBS) and stored at −80°C for future analysis.

### 2.3 Identification of urinary exosomes

In accordance with the identification criteria established by the International Society of Extracellular Vesicles (ISEV), we employed transmission electron microscopy (TEM) to assess the morphology of exosomes. Additionally, nanoparticle tracking analysis (NTA) was utilized to determine the size and concentration of the exosomes, while western blotting was performed to confirm the enrichment of surface proteins associated with these vesicles.

### 2.4 Urine exosome proteomics mass spectrometry analysis

Desalting of the sample was executed using a C18 desalting column. The column was first activated with 100% acetonitrile, followed by equilibration with a 0.1% formic acid solution. After loading the sample onto the column, it was washed with 0.1% formic acid to effectively remove impurities. Elution was carried out using a 70% acetonitrile solution, and the eluate was collected for subsequent lyophilization. The mixed labeled samples were then dissolved in 100 μL of mobile phase A and centrifuged at 14,000 g for 20 min to isolate the supernatant. The sample was fractionated using high-performance liquid chromatography (HPLC) at a flow rate of 0.7 mL/min.

Mobile phase A was composed of 100% water and 0.1% formic acid, while mobile phase B consisted of 80% acetonitrile and 0.1% formic acid. To dissolve the lyophilized powder, we added 10 µL of mobile phase A and centrifuged the mixture at 14,000 g for 20 min at 4°C. A 1-µg sample of the resulting supernatant was injected, and peptides were separated on the analytical column using a linear gradient elution method. An ORBITRAP ECLIPSE mass spectrometer equipped with a Nanospray Flex™ (NSI) ion source was employed, with the ion spray voltage set to 2.0 kV and the ion transfer tube temperature maintained at 320°C. Mass spectrometry was conducted in data-independent acquisition (DIA) mode, covering a full scan range of m/z 350-1,500. For protein-level analysis, a 1% false discovery rate (FDR) filter was applied to the raw mass spectrometry data. Data processing utilized the *Homo sapiens* SP database, and database searching was performed using Spectronaut software.

### 2.5 Bioinformatics analysis

Bioinformatics analysis was performed to interpret the mass spectrometry data. Functional annotation of the identified proteins was conducted using tools such as Gene Ontology (GO) and the Kyoto Encyclopedia of Genes and Genomes (KEGG) to determine their biological roles and pathways. STRING is a database of both known and predicted protein-protein interactions. Probable interacting partners were predicted using the STRING-db server (http://string.embl.de/) based on related species.

### 2.6 Statistical analysis

SPSS 26.0 statistical data processing software (IBM Corp.) was used for analysis, and visualizations were created using GraphPad Prism 8.0 (GraphPad Software Inc.). All experimental data were presented as mean ± standard deviation or median (interquartile range), depending on the nature and distribution of the variables. Differences among continuous variables were analyzed using Student's t-test, the Mann-Whitney U nonparametric test, and ANOVA. Receiver operating characteristic (ROC) curves were plotted to evaluate sensitivity and specificity. All *p*-values were two-tailed, and a *p*-value of less than 0.05 was deemed statistically significant.

## 3 Results

### 3.1 Clinical characteristics

The ages of participants in each group were matched, and no significant differences were observed in the levels of uric acid (UA), total cholesterol (TC), and triglycerides (TG) between the acute gout (AD) group and the indolent gout (ID) group (*P* > 0.05). However, C-reactive protein (CRP) levels were significantly elevated in the AD group *P* < 0.05).

### 3.2 Characteristics of exosomes extracted from urinary samples

**FIGURE 1:**
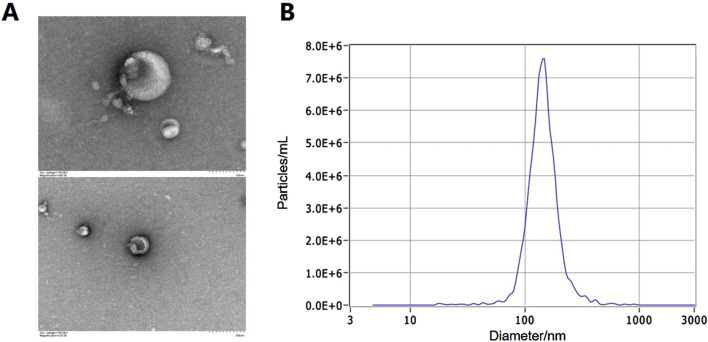
Identification of exosomes in urine. **(A)** The typical bilayer membrane elliptical structures under TEM. **(B)** The result of exosome diameter measurements by NTA.

Exosomes were isolated from urine, and typical bilayer membrane elliptical structures were observed through transmission electron microscopy (TEM), as shown in Figure 1A. The exosomal marker transmembrane protein CD9 was detected through western blot, and nanoparticle tracking analysis (NTA) measurements indicated that the average diameter of the exosomes ranged from 30 to 150 nm, as shown in Figure 1B.

### 3.3 Mass spectrometry analysis of urine exosomes in the AD group and the ID group

**FIGURE 2:**
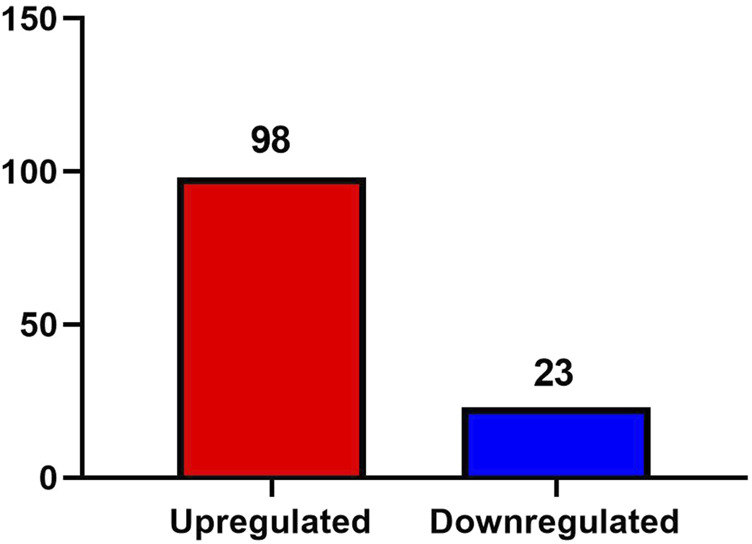
Differentially expressed proteins between the AD group and the ID group.

A total of 1,896 proteins were identified in the urine exosomes of patients from both the AD and ID groups. A fold change greater than 1.5 and a *P* value less than 0.05 were considered significant differences. Compared to the ID group, a total of 121 differentially expressed proteins were identified in the AD group, including 98 proteins that were upregulated and 23 proteins that were downregulated (Figure 2).

### 3.4 Bioinformatics analysis of differentially expressed proteins in urine exosomes

We conducted GO and KEGG analyses on 121 differentially expressed proteins. The biological process analysis revealed that these proteins were closely associated with the NADH metabolic process, negative regulation of the proteasomal ubiquitin-dependent protein catabolic process, and regulation of sodium ion transmembrane transporter activity. The molecular functions primarily involved transmembrane transporter binding, sodium channel regulator activity, and ceramide binding. The cellular components were predominantly located in the cytosol, ficolin-1-rich granule lumen, and extracellular exosomes. The KEGG pathway analysis indicated that these proteins were significantly related to ferroptosis, chemical carcinogenesis (receptor activation), and fatty acid degradation.

### 3.5 Changes in the expression of ferroptosis-related proteins in urine exosomes

**FIGURE 3:**
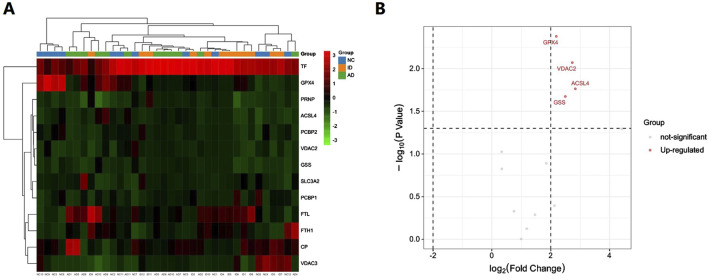
Hierarchical clustering heatmap and the volcano plot of ferroptosis-related proteins in urinary exosomes in the AD group and the ID group. **(A)** The hierarchical clustering heatmap. **(B)** The volcano plot.

Based on the bioinformatics analysis results, we focused on ferroptosis-related proteins. We identified a total of 13 such proteins in the urinary exosomes of patients in both the AD and ID groups (Table 1). The hierarchical clustering heatmap of these proteins is shown in Figure 3A. A fold change greater than 1.5 and a *P*-value less than 0.05 were considered significant. We found that four ferroptosis-related proteins were differentially expressed, all of which were upregulated in the AD group compared to the ID group. The volcano plot illustrating the differentially expressed proteins in urinary exosomes between the AD and ID groups is presented in Figure 3B.

**TABLE 1:** Expression of ferroptosis-related proteins in urinary exosomes in the AD group and the ID group.

UniProt-ID	Gene name	Protein name	FC	*P*-value	Form of expression
O60488	ACSL4	Long-chain-fatty-acid--CoA ligase 4	2.8,363	0.0,172	Up
P45880	VDAC2	Voltage-dependent anion-selective channel protein 2	2.7,324	0.0,085	Up
P48637	GSS	Glutathione synthetase	2.4,947	0.0,212	Up
P36969	GPX4	Phospholipid hydroperoxide glutathione peroxidase GPX4	2.1,894	0.0,042	Up
P00450	CP	Ceruloplasmin	4.4,152	0.0,508	Ns
P02787	TF	Serotransferrin	2.1,264	0.4,028	Ns
P08195	SLC3A2	4F2 cell-surface antigen heavy chain	1.8,484	0.1,286	Ns
P02794	FTH1	Ferritin heavy chain	1.4,657	0.5,143	Ns
P02792	FTL	Ferritin light chain	1.1,868	0.7,509	Ns
Q15365	PCBP1	Poly (rC)-binding protein 1	0.9,960	0.9,893	Ns
Q15366	PCBP2	Poly (rC)-binding protein 2	0.7,514	0.4,691	Ns
Q9Y277	VDAC3	Voltage-dependent anion-selective channel protein 3	0.3,359	0.1,495	Ns
P04156	PRNP	Major prion protein	0.3,335	0.0,943	Ns

### 3.6 Changes in the differentially expression of ferroptosis-related proteins in urine exosomes

**FIGURE 4:**
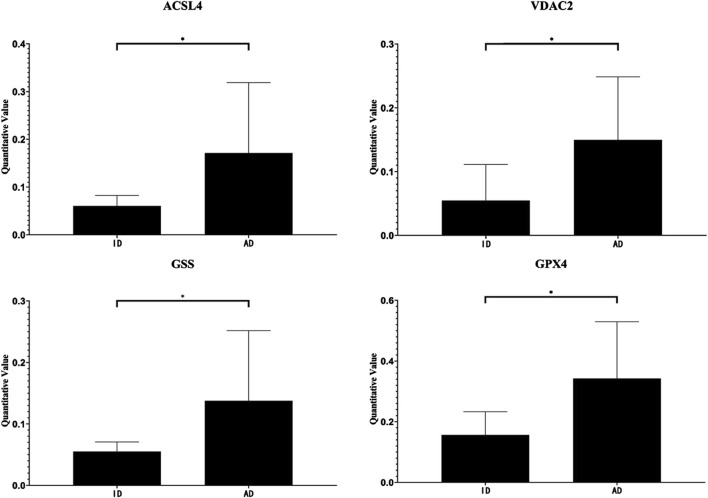
Quantitative value of ACSL, VDAC2, GSS and GPX4. *, *P* < 0.05.

Compared with the ID group, the expression levels of Long-chain fatty-acid–CoA ligase 4 (ACSL4), Voltage-dependent anion-selective channel protein 2 (VDAC2), Glutathione synthetase (GSS), and Phospholipid hydroperoxide glutathione peroxidase GPX4 (GPX4) were elevated in the urinary exosomes of the AD group (Table 1). The quantitative changes of these four differentially expressed proteins between the groups are shown in Figure 4.

### 3.7 Functional analysis of ferroptosis-related proteins in urinary exosomes among AD group and ID group

**FIGURE 5:**
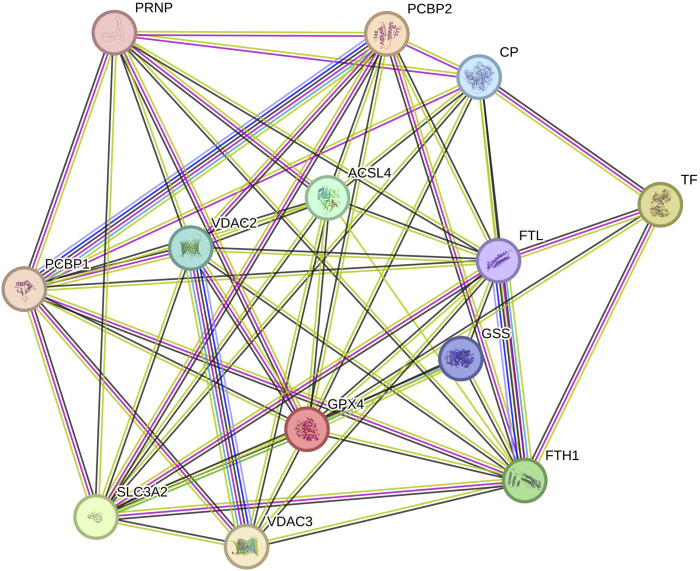
Interactions among 13 ferroptosis-related proteins in urinary exosomes.

We utilized the STRING database to predict interactions among the thirteen ferroptosis-related proteins in urinary exosomes, as illustrated in Figure 5 (more lines indicate stronger correlations). The results demonstrated strong interactions among these proteins, with four differentially expressed proteins occupying more central positions.

**FIGURE 6:**
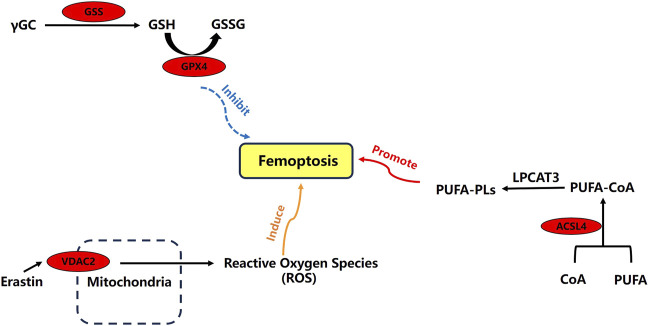
Relationships about four ferroptosis-related differential expression proteins.

Through an extensive literature search, we have mapped out the main mechanisms of ACSL4, VDAC2, GSS, and GPX4 in ferroptosis. These four ferroptosis-related differential expression proteins play critical roles in the process (Figure 6).

### 3.8 Analysis of the clinical value of ferroptosis-related differential expression proteins in urine exosomes for acute gout attack

**FIGURE 7:**
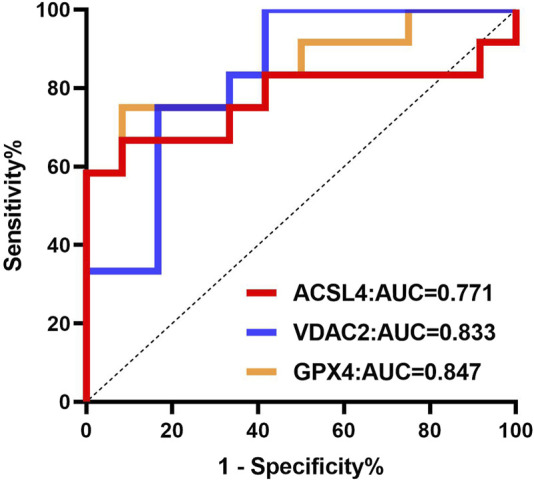
ROC curve analysis of ferroptosis-related differential proteins in urinary exosomes in monitoring of acute gout attack.

The clinical value of ferroptosis-related differential proteins for auxiliary monitoring of acute gout attacks is summarized in Table 2 and Figure 7. In our study, three ferroptosis-related differential proteins showed statistically significant results in ROC analysis. The area under the curve (AUC) for urinary exosome ACSL4 was 0.771 (95% CI, 0.561–0.981), for urinary exosome VDAC2 it was 0.833 (95% CI, 0.668–0.999), and for urinary exosomal GPX4 it was 0.722 (95% CI, 0.687–1.000). In summary, differential proteins related to ferroptosis found in urinary exosomes may serve as potential biomarkers for monitoring acute gout attacks.

**TABLE 2:** Clinical value of ferroptosis-related differential proteins for auxiliary monitoring of acute gout attack.

Ferroptosis-related differential proteins	AUC	95%CI	*P* value	Youden index
ACSL4	0.771	0.561-0.981	0.024	0.583
VDAC2	0.833	0.668-0.999	0.006	0.583
GPX4	0.722	0.687-1.000	0.004	0.667

## 4 Discussion

Gout is an inflammatory arthritis caused by the deposition of monosodium urate (MSU), which is directly related to hyperuricemia resulting from purine metabolism disorders and/or reduced uric acid excretion (Neogi et al., 2015). The incidence of gout is continuously rising worldwide, making it a significant public health issue (Xia et al., 2020). Patients typically experience no symptoms before the acute phase of gout. During a gout attack, the pain can be unbearable, and repeated episodes may lead to damage in multiple organs and tissues. Currently, gout diagnosis requires invasive procedures that can introduce additional pain for patients and do not predict acute attacks. Therefore, the study of non-invasive biomarkers during the acute phase of gout holds significant clinical relevance. Urinary exosomes, which can be collected non-invasively in large quantities, contain valuable clinical information and serve as a promising medium for biomarker research.

Ferroptosis is an iron-dependent form of regulated cell death (Dixon et al., 2012). Its occurrence is linked to the metabolic processes involving iron, amino acids, and lipid peroxides. The fundamental biochemical characteristics of ferroptosis include iron overload, accumulation of intracellular lipid peroxides, and reactive oxygen species (ROS) (Stockwell et al., 2017), and it is closely associated with inflammation (Hassannia et al., 2019). In our study, we identified a total of 1,896 proteins in the urinary exosomes of patients in the AD and ID groups, with 13 proteins associated with ferroptosis. Compared with the ID group, the expression of ACSL4, VDAC2, GSS, and GPX4 in the urinary exosomes of the AD group was significantly increased.

Long-chain acyl-CoA synthetases (ACSLs) are key enzymes responsible for lipid metabolism in the body. The ACSL family includes five isoenzymes: ACSL1, ACSL3, ACSL4, ACSL5, and ACSL6. Among these, ACSL4 plays a role in regulating arachidonic acid (AA) and eicosapentaenoic acid (Chen et al., 2016). Lipid peroxidation is a critical process in ferroptosis, and the upregulation of ACSL4 can lead to lipid peroxidation and induce ferroptosis. Studies have shown that knocking out ACSL4 significantly reduces ferroptosis in mice, thus inhibiting their functional decline and pathological damage (Angeli et al., 2017). Additionally, research indicates that knocking down ACSL4 can decrease inflammatory factors and immune cell infiltration (Wang et al., 2022). VDAC is a transmembrane channel that transports ions and metabolites. There are three isoforms of VDAC: VDAC1, VDAC2, and VDAC3. The ferroptosis inducer erastin can directly bind to VDAC2 and VDAC3, altering the permeability of the mitochondrial outer membrane and reducing the rate of NADH oxidation, thereby inducing ferroptosis. Therefore, VDAC plays a crucial role in regulating this process (DeHart et al., 2018). GPX4, a member of the glutathione peroxidase (GPXs) family, reduces cellular sensitivity to ferroptosis by clearing generated lipid peroxides. If GPX4 generation is blocked or its activity inhibited, the cytotoxic effects from lipid peroxide accumulation cannot be prevented, leading to ferroptosis (Yang et al., 2016). Studies have shown that GPX4 is closely associated with the occurrence and development of gout (Guo et al., 2024). Additionally, GSS catalyzes the synthesis of glutathione (GSH), which is utilized by GPX4 to reduce lipid peroxides. A reduction in GSH synthesis hampers GPX4’s ability to utilize GSH, resulting in ferroptosis (Li et al., 2016). GSH is a vital cofactor for GPX4, helping to clear membrane lipid peroxides and alleviate oxidative stress (Ursini and Maiorino, 2020). It also serves as a major intracellular antioxidant that mitigates inflammation-related cellular damage (Mak et al., 2017). Increased expression of GPX4 can inhibit the oxidation of arachidonic acid (AA) and the activation of the NF-κB pathway, thus reducing ferroptosis and the inflammatory response (Li et al., 2018).

In gout patients, iron overload activates xanthine oxidase and inhibits the expression of ATP-binding cassette subfamily G, member 2 (ABCG2), leading to increased uric acid production and decreased excretion (Furth-Walker and Amy, 1987; Ghio et al., 2002). Intracellular iron overload is key to the execution of ferroptosis (Friedmann Angeli et al., 2019). ACSL4, VDAC2, GSS, and GPX4 are critical proteins in the biological process of ferroptosis. Their increased levels can lead to the accumulation of intracellular lipid peroxides and reactive oxygen species (ROS), which may be closely related to gout, an inflammatory disease. Moreover, ROC analysis in our study indicated that ACSL4, VDAC2, and GPX4 have strong predictive abilities for acute gout attacks, with AUC values of 0.771, 0.833, and 0.722, respectively. These proteins may serve as potential non-invasive biomarkers for monitoring acute gout attacks, providing a foundation for future research and clinical applications in this area.

In conclusion, our study analyzed the expression changes of ferroptosis-related proteins ACSL4, VDAC2, GPX4, and GSS in the urinary exosomes of gout patients. These expression changes may serve as potential non-invasive biomarkers for monitoring acute gout attacks. However, further evaluation and validation with a larger sample size are needed in the future. This study also has certain limitations; thus, in subsequent research, we will expand the sample size to enhance the generalizability of our findings. Additionally, we will validate the key proteins identified through our analysis using other methods.

We employed DIA mass spectrometry combined with bioinformatics to analyze ferroptosis-related proteins in the urinary exosomes of gout patients. This approach aims to evaluate their potential for detecting acute gout attacks and to provide a theoretical basis for non-invasive biomarker research related to the diagnosis and monitoring of acute gout.

## Data Availability

The original contributions presented in the study are included in the article/supplementary material, further inquiries can be directed to the corresponding author.
